# Phase I/II trial of weekly cisplatin, etoposide, and irinotecan chemotherapy for metastatic lung cancer: JCOG 9507

**DOI:** 10.1038/sj.bjc.6600800

**Published:** 2003-03-18

**Authors:** I Sekine, Y Nishiwaki, R Kakinuma, K Kubota, F Hojo, T Matsumoto, H Ohmatsu, K Goto, T Kodama, K Eguchi, T Shinkai, T Tamura, Y Ohe, H Kunitoh, K Yoshimura, N Saijo

**Affiliations:** 1 Internal Medicine and Thoracic Oncology Division, National Cancer Center Hospital, Tsukiji 5-1-1, Chuo-ku, Tokyo 104-0045, Japan; 2 Division of Thoracic Oncology, National Cancer Center Hospital East, Kashiwanoha 6-5-1, Kashiwa 277-8577, Japan; 3 Japan Clinical Oncology Group Data Center, Cancer Information and Epidemiology Division, National Cancer Center Research Institute, Tsukiji 5-1-1, Chuo-ku, Tokyo 104-0045, Japan

**Keywords:** lung cancer, weekly chemotherapy, topoisomerase I inhibitor, topoisomerase II inhibitor

## Abstract

Combinations of cisplatin–irinotecan and cisplatin–etoposide are active and well tolerated in patients with both small-cell lung cancer (SCLC) and nonsmall-cell lung cancer (NSCLC). To define the recommended dose for phase II trials of irinotecan combined with cisplatin and etoposide in chemonaive patients with stage IV disease, 56 patients (11 having SCLC and 45 NSCLC) received cisplatin 25 mg m^−2^ weekly for 9 weeks, etoposide 60 mg m^−2^ for 3 days on weeks 1, 3, 5, 7 and 9, and irinotecan 20–100 mg m^−2^ (levels 1–8) on weeks 2, 4, 6 and 8, together with a prophylactical granulocyte colony-stimulating factor support (50 *μ*g m^−2^ on days 4–7 on weeks 1, 3, 5, 7 and 9, and on days 2–7 on weeks 2, 4, 6 and 8). Grade 3–4 leukocytopenia, neutropenia and thrombocytopenia were noted in 20 (36%), 28 (50%) and nine (16%) patients, respectively. Grade 3 diarrhoea, grade 3 cardiac toxicity, and grade 4 transaminase elevation developed in one (1.8%) patient each. Totally, four of 56 patients were removed from the study because of toxicity and recovered, and two other patients died in situations where drug toxicity might contribute to their death. Dose-limiting toxicity was noted in less than one-third of patients at dose levels 1–7, but in all patients at dose level 8. Thus, the recommended dose was determined to be level 7 (irinotecan 90 mg m^−2^). The response rates for SCLC and NSCLC were 91% (10/11) and 38% (17/45), respectively. The median survival time and 1-year survival rate were 11.9 months and 46% for SCLC and 10.1 months and 40% for NSCLC, respectively. This regimen was considered to be feasible and promising for the treatment of stage IV SCLC and NSCLC.

The combination of cisplatin and etoposide has been the standard chemotherapeutic regimen for small-cell lung cancer (SCLC) and was one of the frequently used regimens in the treatment of nonsmall-cell lung cancer (NSCLC) (Ardizzoni *et al*, 1999; Murren *et al*, 2001). This drug combination has a relatively mild toxicity profile, enabling other cytotoxic agents or thoracic radiotherapy to be added to this regimen (Loehrer *et al*, 1995; Turrisi *et al*, 1999). Irinotecan, a camptothecin derivative topoisomerase I inhibitor, has been shown to exhibit excellent antitumour activity against SCLC and NSCLC in monotherapy and in combination with cisplatin (Fukuoka *et al*, 1992, 2000; Masuda *et al*, 1992; Noda *et al*, 2002). Thus, addition of irinotecan to the cisplatin and etoposide regimen may improve the efficacy against advanced lung cancer.

Weekly chemotherapy regimens have been developed to incorporate multiple drugs into one regimen, to obtain the optimal schedule of each drug, or to increase the dose intensity of cytotoxic agents. A CODE regimen, in which cisplatin, etoposide, doxorubicin and vincristine are administered on a weekly basis for nine cycles, has produced high response rates for both SCLC (85%) and NSCLC (62%) (Murray *et al*, 1991, 1999). A randomised trial of this regimen with and without granulocyte colony-stimulating factor (G-CSF) showed that the addition of G-CSF increased the actual dose intensity of all drugs with a significant improvement in survival (Fukuoka *et al*, 1997). We showed the CODE regimen with the G-CSF support to be highly effective aganist extensive SCLC and relapsed SCLC (Kubota *et al*, 1997; Furuse *et al*, 1998). Thus, although toxicity of the original CODE regimen was greater than that of the standard regimen (Murray *et al*, 1999), the CODE regimen with the G-CSF support is thought to be promising for the treatment of SCLC.

The CODE regimen, in spite of the addition of doxorubicin and vincristine, keeps the dose intensity of cisplatin and etoposide at levels that are comparable to those used in the standard cisplatin and etoposide regimen, which is repeated every 3 weeks (Figure 1

**Figure 1:**
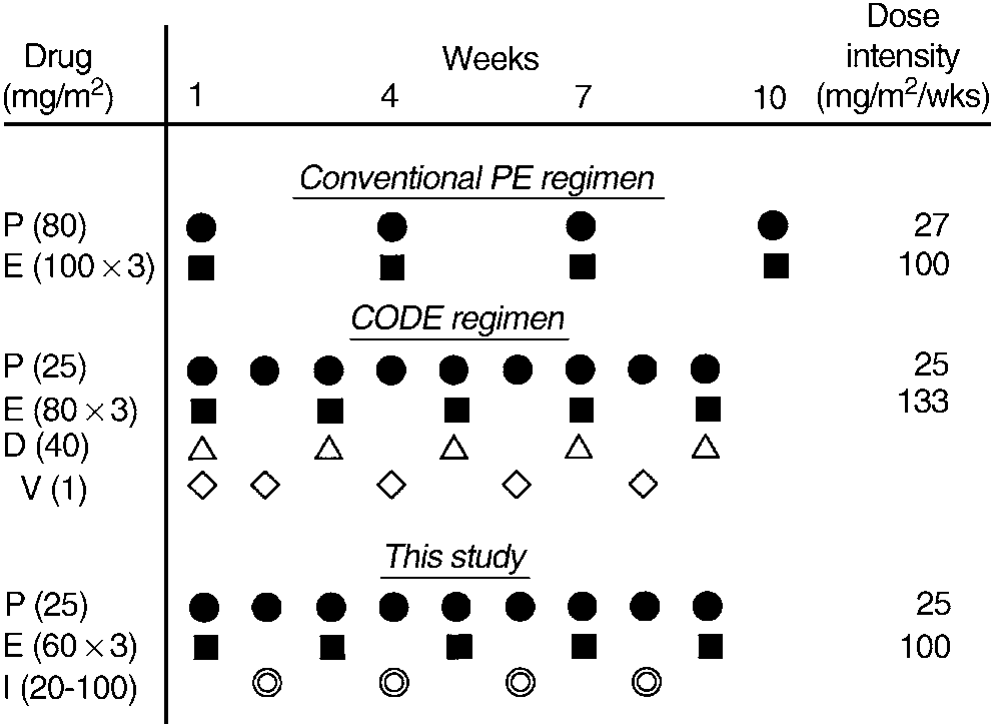
Treatment schedule and dose intensity for the standard cisplatin and etoposide regimen, CODE regimen, and the present study. D (▵): doxorubicin; E (▪): etoposide; I (⦾): irinotecan; P (•): cisplatin; V (⋄): vincristine.

). Thus, it is reasonable to assume that weekly cisplatin and etoposide can be safely combined with another cytotoxic agent by replacing the doxorubicin and vincristine in the CODE regimen with the third agent. Furthermore, this weekly schedule may be of great advantage to obtain synergistic effects of etoposide (topoisomerase II inhibitor) and irinotecan, because the development of resistance to topoisomerase II inhibitors was reported to increase tumour sensitivity to subsequent treatment with topoisomerase I inhibitors (Vasey and Kaye, 1997).

The objectives of this study were: (1) to establish the maximum tolerated dose (MTD) and recommended dose for phase II trials of irinotecan combined with weekly cisplatin and etoposide treatmetns, and (2) to observe the antitumour activity of this regimen in patients with SCLC and NSCLC.

## PATIENTS AND METHODS

### Patient selection

Patients were enrolled in the study if they met the following criteria: (1) a histologic or cytologic diagnosis of lung cancer; (2) metastatic disease (stage IV); (3) age of 70 years or younger; (4) predicted life expectancy of 12 weeks or longer; (5) performance status of 0 or 1 on the Eastern Cooperative Oncology Group (ECOG) scale; (6) no prior chemotherapy; (7) no prior radiotherapy to the primary site; (8) adequate organ function as documented by a WBC count ⩾4.0 × 10^9^ l^−1^, haemoglobin ⩾9.0 g dl^−1^, platelet count ⩾100 × 10^9^ l^−1^, total serum bilirubin ⩽1.5 mg dl^−1^, hepatic transaminases 2 × the normal institutional upper limit of normal or lower, serum creatinine ⩽1.5 mg dl^−1^; and (9) written informed consent.

Patients were not eligible for the study if they had experienced any of the following events: (1) pleural effusion requiring drainage; (2) prior radiotherapy with an irradiated area larger than one-third of the bone marrow volume; (3) synchronous active malignancies other than multiple lung cancers; (4) active infection; (5) contraindications for the use of irinotecan, including diarrhoea, ileus, interstitial pneumonitis, lung fibrosis or massive ascites; (6) serious concomitant medical illness, including severe heart disease, uncontrollable diabetes mellitus or hypertension; or (7) pregnancy or lactation.

### Pretreatment evaluation

Pretreatment assessment included a complete blood cell count, differential counts, routine chemistry measurements, creatinine clearance, blood gas analysis, electrocardiogram, lung function test, chest X-rays, chest conventional tomography, chest computed tomographic (CT) scan, brain CT scan or magnetic resonance imaging, abdominal CT scan or ultrasound sonography, radionuclide bone scan, bone X-rays if indicated, and bronchoscopy.

### Treatment schedule

All therapy was given on an in-patient basis. Cisplatin (25 mg m^−2^) was administered intravenously (i.v.) over 60 min on day 1 and at 1-week intervals for 9 weeks; etoposide (60 mg m^−2^) was administered i.v. over 60 min on days 1–3 of weeks 1, 3, 5, 7 and 9; and irinotecan was administered i.v. over 90 min on day 1 on weeks 2, 4, 6 and 8. The dose levels of irinotecan were 20, 40 mg m^−2^, and subsequent increments of 10 mg m^−2^ up to 100 mg m^−2^. Hydration (2000 ml) and granisetron (40 *μ*g kg^−1^) were given on day 1, followed by a 1000 ml infusion on days 2–5. Prophylactically, G-CSF (50 *μ*g m^−2^) was administered on days when the cytotoxic drugs were not given (on days 4–7 on weeks 1, 3, 5, 7 and 9, and on days 2–7 on weeks 2, 4, 6 and 8), unless the WBC count exceeded 10.0 × 10^9^ l^−1^.

### Toxicity assessment and treatment

During the course of treatment, complete blood cell counts and differential counts were analysed twice a week, and routine chemistry measurements and a chest X-ray were performed once a week. Toxicity was graded according to the toxicity criteria of the Japan Clinical Oncology Group (JCOG) (Tobinai *et al*, 1993), a modified version of the NCI common toxicity criteria issued in 1991. Grade 4 neutropenia is defined as <0.5 × 10^9^ l^−1^ and grade 3 neutropenia is defined as ⩾0.5–<1.0 × 10^9^ l^−1^ in the JCOG criteria. The second and subsequent cycles of chemotherapy were delayed for 1 week if one of the following toxicities was noted on day 1: a WBC count <2.0 × 10^9^ l^−1^, a platelet count <75 × 10^9^ l^−1^, a serum creatinine level ⩾2.0 mg dl^−1^, an elevated hepatic transaminase level or total serum bilirubin of grade 2 or higher, diarrhoea of grade 1–2, fever ⩾38°C, or a performance status of 3. The treatment was terminated if the above-mentioned criteria did not disappear in 3 weeks or if one of the following severe nonhaematological toxicity was noted: diarrhoea of grade 2 lasting for more than 1 week, diarrhoea of grade 3, neurotoxicity of grade 3, or drug-induced pneumonitis.

### Dose-limiting toxicity (DLT), MTD, and recommended dose for phase II trials

The DLT was defined as grade 4 neutropenia lasting for 4 days or longer, grade 4 neutropenia associated with infection, grade 4 thrombocytopenia, grade 3 or severer nonhaematological toxicity other than nausea and vomiting, and the termination of treatment because of the above criteria before four cycles of treatment had been completed. Dose escalation was made, in principle, according to the frequency of DLT in the initial four cycles of the therapy at a level, but the toxicity of all cycles was referred to in the determination of the dose level. Six patients were initially enrolled at each dose level. If one or none of them experienced DLT, then the next cohort of patients was treated at the next higher dose level. If two or three of the six patients experienced DLT, then an additional six patients were enrolled at the same dose level, for a total of 12 patients. If three or fewer of these patients experienced DLT, then the next cohort of patients was treated at the next higher dose level. If four or more of the initial six or of the 12 patients experienced DLT, then that level was considered to be the MTD. The numbers of patients in the cohorts were set to be approximately twice the number of patients in standard phase I trials in order to calculate the median dose intensity for each cohort.

The dose intensity of irinotecan (mg m^−2^ week^−1^) was calculated for each patient using the following formula:


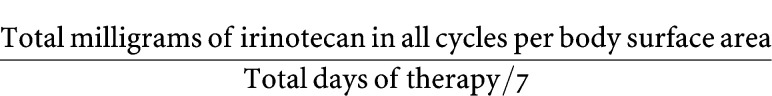


where total days of therapy is the number of days from day 1 of cycle 1 to day 8 of the last cycle (Longo *et al*, 1991). The median dose intensity was then calculated at each dose level. The recommended dose of irinotecan for phase II trials was defined as the dose preceding the MTD. However, the median dose intensity at the dose level was also taken into consideration when determining the recommended dose.

### Response evaluation

Objective tumour responses were evaluated according to the WHO criteria issued in 1979 (WHO, 1979). A complete response (CR) was defined as the disappearance of all known disease for at least 4 weeks with no new lesions appearing. A partial response (PR) referred to an at least 50% decrease of total tumour size for at least 4 weeks without the appearance of new lesions. No change (NC) was defined as the absence of a partial or CR, and no progressive or new lesions were observed for at least 4 weeks. Progressive disease was defined as a 25% or greater increase in the size of any measurable lesion or the appearance of new lesions.

The response rate and its 95% confidence interval for all patients were calculated separately for SCLC and NSCLC in the final analysis. If the upper limit of the interval exceeded 70% in SCLC and 30% in NSCLC, then phase II studies were planned for each tumour type.

### Data management and statistical considerations

This study was designed as a phase I/II trial among two participating centers of the Lung Cancer Study Group in JCOG. The protocol and consent form were approved by the Clinical Trial Review Committee of JCOG and the Institutional Review Board of the National Cancer Center. Patient registration and data management were performed by the JCOG Data Center. Interim monitoring was performed by the JCOG Data Center to ensure quality control. Monitoring reports were submitted to and reviewed by the JCOG Data and Safety Monitoring Committee (DSMC) semiannually. The planned accrual and follow-up periods were 12 and 24 months, respectively. The duration of overall survival was measured from the date of registration to the date of death from any cause or the last follow-up. The survival distribution was estimated using the Kaplan–Meier method, and the confidence intervals were based on Greenwoods' formula (Armitage and Berry, 1994). The statistical analysis was performed mostly by the JCOG Data Center using SAS software version 6.12 for Windows (SAS Institute Inc., Cary, NC, USA). The dose intensity was calculated by the Study Coordinator.

## RESULTS

From June 1995 to December 1997, 56 patients were entered in the study; the last follow-up was performed in December 1999. When six patients were registered at each dose level, the registration was suspended to assess the DLT of patients entered at each dose level. Totally, the registration was closed for 6.6 months. The demographic details are listed in Table 1

**Table 1:** Patient characteristics

	**Median (range)**	**N**	**(%)**
Number of patients		56	
*Gender*			
Male		41	(73)
Female		15	(27)
Age	58 (38–70)		
PS			
0		12	(21)
1		44	(79)
*Body weight loss*			
0%		38	(68)
1–9%		14	(25)
⩾10%		4	(7)
*Histology*			
Small-cell carcinoma		11	(20)
Adenocarcinoma		35	(63)
Squamous-cell carcinoma		5	(9)
Large-cell carcinoma		4	(7)
Others		1	(2)

. All patients were chemonaive, and had stage IV disease. Small-cell carcinoma accounted for 11 (20%) of the 56 patients. One patient enrolled at dose level 5 was treated with etoposide at an erroneous dose of 100 mg m^−2^ for 3 days during the first cycle and at the correct dose during subsequent cycles. This was a major protocol violation, but nine cycles of the treatment were completed without severe toxicity in this patient. Therapy was terminated because of progressive disease in four patients, patient refusal because of the lack of antitumour effect in one patient, toxicity in four patients, and death in one patient. The last patient received three cycles of the therapy, developed grade 4 neutropenia for 7 days (DLT) and leukopenia for 3 days, and died 29 days after the registration of massive haemoptysis from the tumour, which exhibited a large reduction in size and cavitation. Another patient who was treated at dose level 5 developed cardiac tamponade and an elevated hepatic transaminase level of grade 4 (DLT) as a result of a congestive liver, and died 15 days after the completion of nine cycles of the therapy. Cytological analysis of pericardial effusion was not performed in this patient. No other deaths occurred during or within 30 days of the therapy.

### Treatment delivery and dose intensity

The treatment delivery and dose intensity were evaluated in 51 patients (Table 2

**Table 2:** Treatment delivery and dose intensity (*N*=51)

	**Dose of**	**No. of patients**			**Median dose intensity (mg m^−2^ week^−1^)**		
**Level**	**irinotecan(mg m^−2^)**	**Evaluated**	**Completed nine cycles**	**(%)**	**Projected**	**Actual**	**(%)**
1	20	6	5	83	8.6	8.6	99
2	40	6	6	100	17.2	17.2	100
3	50	5	5	100	21.6	21.5	100
4	60	5	4	80	25.9	21.3	82
5	70	6	6	100	30.2	27.2	90
6	80	12	10	83	34.5	32.8	95
7	90	7	7	100	38.8	34.5	89
8	100	4	2	50	43.1	30.4	71

Five patients were excluded from this analysis because of progressive disease in four patients and protocol violation in one patient.

). Five patients were excluded from the analysis, because of the early discontinuation of this therapy as a result of progressive disease in four patients and a protocol violation in one patient. At dose levels 1–7, 80% or more of the patients completed all nine cycles of the therapy, whereas only 50% of the patients at dose level 8 completed the regimen. The percentages of actual median dose intensity against projected dose intensity were more than 80% at dose levels 1–7 but only 71% at dose level 8. The dose level that produced the highest dose intensity of irinotecan (34.5 mg m^−2^ week^−1^) was level 7.

### Toxicity, MTD, and recommended dose for phase II trials

Severe toxicity was mainly haematological. Grade 3–4 leukopenia, neutropenia and thrombocytopenia were observed in 20 (36%), 28 (50%), and nine (16%) of the 56 patients, but no febrile neutropenia was encountered. Grade 3 diarrhoea (DLT) was noted in only one patient at dose level 6 (Table 3

**Table 3:** Toxicity evaluated for all cycles (*N*=56)

**Grade**	**Absent**	**1**	**2**	**3**	**4**
Anaemia	0	2	19	29	—
Leukocytopenia	0	13	18	17	3
Neutropenia	0	5	15	17	11
Thrombocytopenia	0	5	11	8	1
Elevated total bilirubin	0	—	14	0	0
Elevated GOT	0	16	0	1	0
Elevated GPT	0	27	3	0	1
Elevated creatinine	0	6	3	0	0
Hyponatremia	0	21	6	1	0
Hypokalemia	0	10	2	1	0
Infection	0	8	6	0	0
Nausea/vomiting	1	26	19	4	—
Diarrhoea	0	21	6	1	0
Stomatitis	1	6	1	0	0
Alopecia	2	37	13	0	—

). One patient at dose level 8 complained of chest pain after the completion of two cycles of therapy. The patient had a history of angina pectoris and had undergone a percutaneous transluminal coronary angioplasty 7 months before the cancer treatment. The electrocardiogram during the attack showed an elevated ST segment at the II, III, and aVF leads, but the following electrocardiograms were normal. A coronary artery angiography performed on the same day revealed no narrowing in the coronary artery, but the administration of ergotamine induced vasospasm in the artery. A diagnosis of variant angina was made, and therefore there was possibility that coronary artery spasm was induced by the administration of anticancer agents. This was counted as grade 3 cardiac toxicity (DLT). Nonhaematologic toxicity was mild and transient in all other patients.

DLT occurred in less than one-third of patients treated at dose levels 1–7, whereas DLT developed within four cycles of therapy in two patients treated at dose level 8, and within nine cycles in all patients treated at this level (Table 4

**Table 4:** Dose-limiting toxicity by dose levels (*N*=56)

		**No. of pts with DLT within**		
**Level**	**Total no. of pts**	**Four cycles**	**All cycles**	**Type of DLT (no. of pts)**
1	6	0	0	None
2	6	0	0	None
3	7	0	0	None
4	6	1	1	Neutropenia+hyponatremia (1)
5	7	0	2	Neutropenia+hypokalemia (1), elevated hepatic transaminases (1)
6	13	2	4	Neutropenia (2), thrombocytopenia (1), diarrhoea (1)
7	7	0	1	Neutropenia (1)
8	4	2	4	Neutropenia (3), cardiac ischaemia (1)

DLT: dose-limiting toxicity; pts: patients.

). Thus, the MTD was determined to be dose level 8. In consideration of the good treatment delivery and the highest dose intensity achieved at dose level 7, the recommended dose for the phase II trials was established to be dose level 7, or 90 mg m^−2^ of irinotecan, for this schedule.

### Objective responses and survival

All the patients were included in the analyses of tumour response and survival. The response rate (95% confidence interval) was 91% (59–100%) in 11 patients with SCLC and 38% (24–54%) in 45 patients with NSCLC (Table 5

**Table 5:** Tumour responses according to histologic type (*N*=56)

	**Small-cell carcinoma**		**Nonsmall-cell carcinoma**	
**Response**	***N***	**(%)**	***N***	**(%)**
Complete response	2	(18)	0 (0)	
Partial response	8	(73)	17	(38)
No change	0	(0)	18	(40)
Progressive disease	0	(0)	8	(18)
Not evaluable	1	(9)	2	(4)

). The response rate in the 23 NSCLC patients registered at dose levels 1–5 was 39%, while that in the 22 NSCLC patients registered at dose levels 6–8 was 36%. The median survival time (95% confidence interval) and 1-year survival rate (95% confidence interval) were 11.9 (9.7–15.6) months and 46% (16–75%) in the 11 patients with SCLC and 10.1 (7.8–12.3) months and 40% (26–54%) in the 45 patients with NSCLC, respectively.

## DISCUSSION

This study established the recommended dose of irinotecan (90 mg m^−2^ biweekly) for phase II studies in combination with weekly cisplatin (25 mg m^−2^ weekly) and etoposide (60 mg m^−2^ for 3 days biweekly) treatments and a G-CSF support. At this dose level, nine weekly consecutive administrations of chemotherapy were completed in more than 80% of patients, and the highest dose intensity was obtained. The cumulative doses of cisplatin and irinotecan at their recommended doses, 225 and 360 mg m^−2^, respectively, corresponded to 94 and 50%, respectively, of those in the standard cisplatin and irinotecan regimen, which is repeated every 4 weeks for a total of four cycles. The dose intensity of irinotecan, however, was 86% of that in the standard regimen (Noda *et al*, 2002). The cumulative dose of etoposide is 75% of that in the standard cisplatin and etoposide regimen repeated every 3 weeks for a total of four cycles, but the dose intensity of etoposide is the same as that in the standard regimen (Noda *et al*, 2002). Thus, although the cumulative doses of irinotecan and etoposide are less than those for the standard regimens, the dose intensity of each drug is well maintained and comparable to those of the standard regimens.

Some of the criteria in this study's protocol were determined arbitrarily because of the lack of published reports on dose findings for weekly chemotherapy regimens. First, the decision to escalate the dose was made when the toxicity and DLT data for the initial four cycles of therapy had been collected. This protocol was adopted because (1) DLT is generally assessed after one or two cycles of conventional chemotherapy repeated every 3–4 weeks, and this duration was considered to correspond to four cycles of our regimen, and (2) we felt that too much time was required to complete an evaluation of toxicity for all cycles. The registration period in this study was 2.5 years, 2.5 times longer than planned, but the total duration of the pause in registration required to evaluate DLT was not as long as expected. Second, the recommended dose for phase II trials was defined in two ways according to the protocol of the current study: the dose level prior to MTD and the dose producing the highest dose intensity. The doses determined by the two definitions were consistent in this study, but they could differ for other chemotherapy combinations. Which definition is preferable depends largely on how important it is to increase the dose intensity of the chemotherapeutic regimen. Third, this study required a large number of patients. The number of patients required at each dose level to calculate the dose intensity was 6–12, twice as many as a conventional phase I trial. This method of calculation, however, would be sufficient at dose levels close to the recommended dose. In addition, the starting dose of irinotecan, which was determined to be 20 mg m^−2^, might have been too low. Standard criteria for the selection of a starting dose in combination phase I studies have not been established.

This is the first study to combine the use of the topoisomerase I inhibitor irinotecan and the topoisomerase II inhibitor etoposide together with cisplatin. Topoisomerases are essential for the maintenance of cellular viability throughout the cell cycle, and alterations in the regulation of one topoisomerase are often compensated for by alterations in the other. Preclinical studies showed that cell lines with deficient topoisomerase I activity were more sensitive to topoisomerase II inhibitors, and that cell lines resistant to topoisomerase II inhibitors have increased sensitivity to topoisomerase I inhibitors (Vasey and Kaye, 1997). In clinical trials, however, concurrent or sequential administration of irinotecan and etoposide resulted in severe toxicity and failed to produce a superior antitumour activity than irinotecan alone in patients with NSCLC (Ando *et al*, 1997; Oshita *et al*, 1997). In the present trial, each agent was given separately for about 1 week. The relatively mild toxic profile of this study may be partly attributable to the separate administration of these two agents.

The response rates and their confidence intervals for SCLC and NSCLC in this study exceeded the criteria required for a phase II study. The median survival times of 11.9 and 10.1 months for metastatic SCLC and NSCLC, respectively, were also promising, although the number of patients with SCLC was too small to evaluate survival. We are conducting two phase II studies, one in patients with untreated extensive SCLC and the other in patients with recurrent SCLC. We have not planned a phase II study for NSCLC, because several new promising agents for this disease are now available.

In conclusion, this is the first study to define a schedule for a triplet combination of cisplatin, irinotecan and etoposide with a G-CSF support. The recommended dose of irinotecan for further studies is 90 mg m^−2^ repeated every 2 weeks in combination with weekly cisplatin and biweekly etoposide treatments. This treatment regimen shows promising antitumour activity in patients with stage IV SCLC and NSCLC.

## References

[bib1] Ando M, Eguchi K, Shinkai T, Tamura T, Ohe Y, Yamamoto N, Kurata T, Kasai T, Ohmatsu H, Kubota K, Sekine I, Hojo N, Matsumoto T, Kodama T, Kakinuma R, Nishiwaki Y, Saijo N (1997) Phase I study of sequentially administered topoisomerase I inhibitor (irinotecan) and topoisomerase II inhibitor (etoposide) for metastatic non-small-cell lung cancer. Br J Cancer 76: 1494–1499 9400948 10.1038/bjc.1997.584 PMC2228187

[bib2] Ardizzoni A, Antonelli G, Grossi F, Tixi L, Cafferata M, Rosso R (1999) The combination of etoposide and cisplatin in non-small-cell lung cancer (NSCLC). Ann Oncol 10(Suppl 5): S13–S17 10582133 10.1093/annonc/10.suppl_5.s13

[bib3] Armitage P, Berry G (1994) Survival Analysis. In Statistical Methods in Medical Research, 3rd edn, Armitage P, Berry G (eds) pp 469–492. Oxford: Blackwell Scientific Publications

[bib4] Fukuoka M, Masuda N, Negoro S, Matsui K, Yana T, Kudoh S, Kusunoki Y, Takada M, Kawahara M, Ogawara M, Kodama N, Kubota K, Furuse K (1997) CODE chemotherapy with and without granulocyte colony-stimulating factor in small-cell lung cancer. Br J Cancer 75: 306–309 9010043 10.1038/bjc.1997.50 PMC2063260

[bib5] Fukuoka M, Nagao K, Ohashi Y, Niitani H (2000) Impact of irinotecan (CPT-11) and cisplatin (CDDP) on survival in previously untreated metastatic non-small cell lung cancer (NSCLC). Proc Am Soc Clin Oncol 19: 495a

[bib6] Fukuoka M, Niitani H, Suzuki A, Motomiya M, Hasegawa K, Nishiwaki Y, Kuriyama T, Ariyoshi Y, Negoro S, Masuda N (1992) A phase II study of CPT-11, a new derivative of camptothecin, for previously untreated non-small-cell lung cancer. J Clin Oncol 10: 16–20 1309380 10.1200/JCO.1992.10.1.16

[bib7] Furuse K, Fukuoka M, Nishiwaki Y, Kurita Y, Watanabe K, Noda K, Ariyoshi Y, Tamura T, Saijo N (1998) Phase III study of intensive weekly chemotherapy with recombinant human granulocyte colony-stimulating factor *versus* standard chemotherapy in extensive-disease small-cell lung cancer. The Japan Clinical Oncology Group. J Clin Oncol 16: 2126–2132 10.1200/JCO.1998.16.6.2126 9626212

[bib8] Kubota K, Nishiwaki Y, Kakinuma R, Hojo F, Matsumoto T, Ohmatsu H, Sekine I, Yokozaki M, Goto K, Ebi N, Kodama T (1997) Dose-intensive weekly chemotherapy for treatment of relapsed small-cell lung cancer. J Clin Oncol 15: 292–296 8996155 10.1200/JCO.1997.15.1.292

[bib9] Loehrer Sr PJ, Ansari R, Gonin R, Monaco F, Fisher W, Sandler A, Einhorn LH (1995) Cisplatin plus etoposide with and without ifosfamide in extensive small-cell lung cancer: a Hoosier Oncology Group study. J Clin Oncol 13: 2594–2599 7595712 10.1200/JCO.1995.13.10.2594

[bib10] Longo DL, Duffey PL, DeVita Jr VT, Wesley MN, Hubbard SM, Young RC (1991) The calculation of actual or received dose intensity: a comparison of published methods. J Clin Oncol 9: 2042–2051 1941063 10.1200/JCO.1991.9.11.2042

[bib11] Masuda N, Fukuoka M, Kusunoki Y, Matsui K, Takifuji N, Kudoh S, Negoro S, Nishioka M, Nakagawa K, Takada M (1992) CPT-11: a new derivative of camptothecin for the treatment of refractory or relapsed small-cell lung cancer. J Clin Oncol 10: 1225–1229 1321891 10.1200/JCO.1992.10.8.1225

[bib12] Murray N, Livingston RB, Shepherd FA, James K, Zee B, Langleben A, Kraut M, Bearden J, Goodwin JW, Grafton C, Turrisi A, Walde D, Croft H, Osoba D, Ottaway J, Gandara D (1999) Randomized study of CODE *versus* alternating CAV/EP for extensive-stage small-cell lung cancer: an Intergroup Study of the National Cancer Institute of Canada Clinical Trials Group and the Southwest Oncology Group. J Clin Oncol 17: 2300–2308 10561291 10.1200/JCO.1999.17.8.2300

[bib13] Murray N, Osoba D, Shah A, Page R, Karsai H, Little C (1991) Brief intensive chemotherapy for metastatic non-small-cell lung cancer: a phase II study of the weekly CODE regimen. J Natl Cancer Inst 83: 190–194 1846431 10.1093/jnci/83.3.190

[bib14] Murren J, Glatstein E, Pass HI (2001) Small cell lung cancer. In Cancer: Principles & Practice of Oncology, 6th edn. DeVita Jr VT, Hellman S, Rosenberg SA (eds) pp 983–1018. Philadelphia: Lippincott Williams & Wilkins

[bib15] Noda K, Nishiwaki Y, Kawahara M, Negoro S, Sugiura T, Yokoyama A, Fukuoka M, Mori K, Watanabe K, Tamura T, Yamamoto S, Saijo N (2002) Irinotecan plus cisplatin compared with etoposide plus cisplatin for extensive disease small-cell lung cancer. N Engl J Med 346: 85–91 11784874 10.1056/NEJMoa003034

[bib16] Oshita F, Noda K, Nishiwaki Y, Fujita A, Kurita Y, Nakabayashi T, Tobise K, Abe S, Suzuki S, Hayashi I, Kawakami Y, Matsuda T, Tsuchiya S, Takahashi S, Tamura T, Saijo N (1997) Phase II study of irinotecan and etoposide in patients with metastatic non-small-cell lung cancer. J Clin Oncol 15: 304–309 8996157 10.1200/JCO.1997.15.1.304

[bib17] Tobinai K, Kohno A, Shimada Y, Watanabe T, Tamura T, Takeyama K, Narabayashi M, Fukutomi T, Kondo H, Shimoyama M (1993) Toxicity grading criteria of Japan Clinical Oncology Group. Jpn J Clin Oncol 23: 250–257 8411739

[bib18] Turrisi 3rd AT, Kim K, Blum R, Sause WT, Livingston RB, Komaki R, Wagner H, Aisner S, Johnson DH (1999) Twice-daily compared with once-daily thoracic radiotherapy in limited small-cell lung cancer treated concurrently with cisplatin and etoposide. N Engl J Med 340: 265–271 9920950 10.1056/NEJM199901283400403

[bib19] Vasey PA, Kaye SB (1997) Combined inhibition of topoisomerases I and II – is this a worthwhile/feasible strategy? Br J Cancer 76: 1395–1397 9400932 10.1038/bjc.1997.568 PMC2228166

[bib20] WHO (1979) Handbook for Reporting Results of Cancer Treatment. WHO Offset Publication No. 48. Geneva: World Health Organization

